# Chronic Arsenic Exposure and Risk of Post Kala-azar Dermal Leishmaniasis Development in India: A Retrospective Cohort Study

**DOI:** 10.1371/journal.pntd.0005060

**Published:** 2016-10-24

**Authors:** Sushmita Das, Rakesh Mandal, Vidya Nand Rabidas, Neena Verma, Krishna Pandey, Ashok Kumar Ghosh, Sreekant Kesari, Ashish Kumar, Bidyut Purkait, Chandra Sekhar Lal, Pradeep Das

**Affiliations:** 1 Department of Microbiology, All-India Institute of Medical Sciences, Patna, Bihar, India; 2 Department of Vector Biology, Rajendra Memorial Research Institute of Medical Sciences, Indian Council of Medical Research (I.C.M.R.), Patna, Bihar, India; 3 Department of Clinical Medicine, Rajendra Memorial Research Institute of Medical Sciences, Indian Council of Medical Research (I.C.M.R.), Patna, Bihar, India; 4 Department of Pathology, Rajendra Memorial Research Institute of Medical Sciences, Indian Council of Medical Research (I.C.M.R.), Patna, Bihar, India; 5 Department of Environment and Water Management, A.N. College, Patna, Bihar, India; Hebrew University-Hadassah Medical School, ISRAEL

## Abstract

**Background:**

Visceral leishmaniasis (VL), with the squeal of Post-kala-azar dermal leishmaniasis (PKDL), is a global threat for health. Studies have shown sodium stibogluconate (SSG) resistance in VL patients with chronic arsenic exposure. Here, we assessed the association between arsenic exposure and risk of developing PKDL in treated VL patients.

**Methods:**

In this retrospective study, PKDL patients (n = 139), earlier treated with SSG or any other drug during VL, were selected from the study cohort. Trained physicians, unaware of arsenic exposure, interviewed them and collected relevant data in a questionnaire format. All probable water sources were identified around the patient’s house and water was collected for evaluation of arsenic concentration. A GIS-based village-level digital database of PKDL cases and arsenic concentration in groundwater was developed and individual point location of PKDL cases were overlaid on an integrated GIS map. We used multivariate logistic regression analysis to assess odds ratios (ORs) for association between arsenic exposure and PKDL development.

**Results:**

Out of the 429 water samples tested, 403 had arsenic content of over 10 μg/L, with highest level of 432 μg/L among the seven study villages. Multivariate adjusted ORs for risk of PKDL development in comparison of arsenic concentrations of 10.1–200 μg/L and 200.1–432.0 μg/L were 1.85 (1.13–3.03) and 2.31 (1.39–3.8) respectively. Interestingly, similar results were found for daily dose of arsenic and total arsenic concentration in urine sample of the individual. The multivariate-adjusted OR for comparison of high baseline arsenic exposure to low baseline arsenic exposure of the individuals in the study cohort was 1.66 (95% CI 1.02–2.7; p = 0.04).

**Conclusion:**

Our findings indicate the need to consider environmental factors, like long time arsenic exposure, as an additional influence on treated VL patients towards risk of PKDL development in Bihar.

## Author Summary

Post-kala-azar dermal leishmaniasis (PKDL) is a sequela of visceral leishmaniasis (VL) that appears after patients have apparently been cured of visceral leishmaniasis; even been reported in patients without a history of VL. Previous clinical and epidemiological data ascertains the main risk factor associated with the development of PKDL is previous treatment for VL with antimonials (SSG); however, PKDL also occurs after treatment with other drugs like paromomycin, miltefosine etc. Here, in light of the risk of arsenic-associated dermal manifestations, we hypothesized that the long term exposure to groundwater arsenic acts as an additional risk factor for development of PKDL in patients treated for VL with SSG or other drugs. Using a cohort, we retrospectively assessed the risk of arsenic in development of PKDL in treated VL patients. Our findings support a significant association and prompts parasites might persist successfully in individuals over-exposed to arsenic and may exhibit features of dermatotropism leading to development of PKDL after treatment for VL. Further research is needed to dissect the mechanistic role of arsenic on VL, as well as PKDL development.

## Introduction

Visceral Leishmaniasis (VL) is one of the neglected tropical diseases and a major global threat for health worldwide [1,2]. It poses a major health problem in the poverty-ridden state of Bihar, which accounts for nearly 90% of the total cases in India [3]. Post-kala-azar dermal leishmaniasis (PKDL), a dermal sequel of VL, is caused by *L*. *donovani*, and is confined to South Asia (India, Nepal, and Bangladesh) and East Africa, mainly Sudan [4,5]. In Africa, papular or nodular lesions are seen whereas in South Asia, the disease is mainly represented by polymorphic lesions with macules and/or with papulonodules [4]. However, the incidence of PKDL varies from 5–10% in India (http://www.who.int/leishmaniasis/resources/INDIA.pdf) and to 50–60% in Sudan [6, 7]. In many aspects, asian PKDL is much different from the African counterpart [5]. African PKDL may exhibit spontaneous cure, co-occur with the visceral disease and even develop in VL patients undergoing treatment; which is not the case for Indian PKDL. Indian PKDL is characterized by appearance of hypopigmented macular, papular and/or nodular rashes on the skin; often represented by erythema and induration on the face [6, 7]. PKDL is a perplexing disease and its role in transmission of VL warrants much debate. Risk factors for development of PKDL need extensive study as the control is an inevitable part of the current worldwide VL elimination programme.

Groundwater is an essential component of our water resources for drinking, irrigation and industrial purposes. There is growing concern on deterioration of ground water quality due to geogenic and anthropogenic activities. Ground water contains wide varieties of dissolved inorganic chemical constituents in various concentrations due to chemical and biochemical interactions between water and the geological materials through contribution from the atmosphere and surface water bodies. Arsenic (As) is a colorless and odorless toxic metalloid element present in airborne particles, water, food and soil; and presents serious human health hazard [8]. Inorganic arsenic tends to be more toxic than organic arsenic. Arsenic exposure can result into chronic arsenic toxicity (CAT), hence, long-term exposure is associated with diabetes, skin disease, various types of cancers, chronic cough, and toxic effects in the liver, kidney, cardiovascular system, and the peripheral and central nervous systems [9]. Importantly, CAT are mainly known to result in skin lesions and various systemic manifestations like chronic lung diseases (chronic bronchitis, chronic obstructive pulmonary disease and bronchiectasis), liver diseases (non-cirrhotic portal fibrosis) and other diseases like peripheral vascular disease, polyneuropathy, hypertension and ischeamic heart disease, oedema, diabetes mellitus, weakness and even anaemia [10,11]. Among all, dermal effects following the exposure to arsenic are hallmarks of arsenic poisoning [12, 13]. Chronic arsenic exposure leads to the development of skin lesions, including hyperkeratosis and hyperpigmentation [14]. CAT-induced keratosis emerges as diffuse thickening of palms and soles, alone or in combination with nodules, and are usually symmetrically distributed.

Safe drinking water is a fundamental human right and one of the basic needs of an individual. Arsenic contamination of drinking groundwater has majorly impacted environmental health throughout the world including India [15,16]. Arsenic is a widely dispersed element in the Earth's crust and principally exists in the environment as sulfides, oxides, and phosphates. There are many possible routes of human exposure to arsenic from both natural and anthropogenic sources [9]. Groundwater contamination by arsenic arises from sources of arsenopyrite, base metal sulfides, realgar (arsenic-sulfide mineral) and orpiment, arsenic-rich pyrite, and iron oxyhydroxide. Arsenic is released from minerals by many bio-geochemical processes: oxidation of arsenic-bearing sulfides, desorption from oxides and hydroxides, reductive dissolution, evaporative concentration, leaching from sulfides by carbonate, and microbial mobilization [15, 16]. Notably, arsenopyrite is a relatively soluble mineral with pH conditions typically found in groundwater (pH 6.5–8.5) and both in oxidizing and slightly reduced environments (i.e., Eh values greater than approximately −0.2 V) [16]. It breaks down to liberate mobile arsenic species, such as arsenite and arsenate, along with iron and sulphur into surface water and groundwater, thus contaminating the water supplies [17]. Rapid industrialization, ineffective water purification and sewage management systems, periodic monsoon and flooding etc are the main players that have exacerbated the problem of arsenic groundwater contamination in India since the past four decades [18,19].

Arsenic poisoning is a medical condition caused by elevated levels of sodium arsenite in different parts of the body. In recent years, it has assumed an alarming proportion in different parts of the Gangetic plain (Uttar Pradesh, Bihar and West Bengal), where growing arsenic contamination of drinking water has been reported [18–24]. Notably, the problem of groundwater contamination in 18 out of the 38 districts in Bihar has reached alarming proportions since arsenic in groundwater was first reported in June 2002 in Bhojpur district [22–24]. Arsenic contamination up to 1861 ppb was found in regions of Bihar, against the W.H.O. permissible limit of 10 ppb. Undoubtedly, enhancement of arsenic in cultivated land by irrigation with contaminated water accelerate the uptake level through consumption of agricultural products such as rice, vegetables and other food crops; which have been one of the major cause of the increasing number of health issues in the affected regions [25–27]. Currently, exposure to arsenic has been supposedly linked with development of antimonial resistance among VL patients in Bihar [28,29], as arsenic shares common chemical properties with antimony [28]. Antimonial resistance in VL has also been linked with arsenic exposure and development of cross-resistance has been suggested leading to the enhanced power of the parasites to survive during SSG treatment. Exposure to arsenic toxicity over decades, is thought to be responsible for disrupting pathways of antimony action on the parasite resulting in antimony resistance [29]. Reportedly, development of PKDL has been strongly linked with administration of SSG, for treatment of VL [30]. Epidemiological data and clinical reports have also strongly supported this link [29]. Simultaneously, the prime manifestations of CAT are skin lesions, characterized by pigmentation and keratosis [11]. However, development of PKDL in VL patients treated with other drugs, viz. paromomycin, amphotericin, miltefosine etc, have also been reported [30]. Therefore, we hypothesized of the contributing role for some environmental factors, like chronic exposure of arsenic contaminated groundwater, in development of PKDL. In this study, in light of the risk of CAT-associated dermal manifestations, we hypothesized that the long term exposure to groundwater arsenic contamination acts as a risk factor for development of PKDL in patients treated for VL. This was a retrospective study, as the time period between VL and development of PKDL varied among patients. As previous reports suggest prevalence of groundwater arsenic contamination in the gangetic plains [11, 22–25], we sought to test our hypothesis in the Raghopur cohort of Vaishali district in Bihar, which is mostly surrounded by the Ganges from all sides.

## Methods

### Ethics statement

This research was conducted with approval of the institutional human Ethical Committee (IHEC) and conducted as per the guidelines of the Institutional Review Board of Rajendra Memorial Research Institute of Medical Sciences (ICMR), Patna, India. All PKDL patients provided written informed consent for participation in this research study and subsequent analysis. Groundwater arsenic contamination was reported to UNICEF and the arsenic mitigation team, Public Health and Education Department, Bihar.

### The study cohort

**Fig 1:**
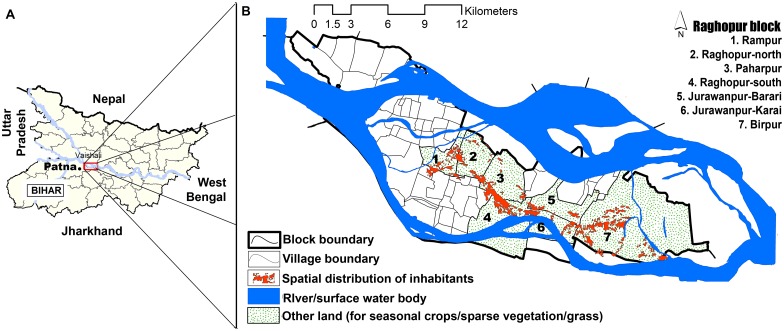
Map of the study cohort. A. Map of Bihar state, India with the boxed study cohort. B. Detailed map of Raghopur block showing the study cohort with seven villages of interest; details of water bodies, land and inhabitant distribution.

Being totally intercepted by the Ganges, we selected Raghopur block of the Vaishali District, Bihar, as the target cohort for our retrospective study (Fig 1). The Raghopur block is located in the southern part of the district, mainly surrounded by some kala-azar endemic blocks namely Hajipur, Bidupur, Shadei Buzurg in north and Mahanar in the eastern side.The block also shares its border with Patna district in the south and Saran district to the west. A total of seven villages, namely Birpur, Jurawanpur Barari, Jurawanpur Karai, Paharpur, Raghopur-north, Raghopur-south and Rampur, were selected in the Raghopur block for this study. These villages are known to be highly endemic for VL with yearly average case incidence of 7.81, 6.33, 11.93, 7.40, 8.02, 4.65 and 1.46 per 10,000 population in Birpur, Jurawanpur Barari, Jurawanpur Karai, Paharpur, Raghopur-north, Raghopur-south and Rampur respectively, during 2012–2014 [Source: District Malaria Office Hajipur (DMOH), Vaishali and State Health Society Bihar (SHSB), Patna]. The area is flat and covered by alluvium of still clay and sand deposited by the Ganges (Fig 1). Most of the land areas are frequently interrupted by river tributaries and small ground surface water collections. Sparse vegetation canopy existed in the study area that mainly includes grass land and plantations of mainly banana and mango. Cropping pattern is dominated by cereals such as rice, wheat and maize. Climate of the study area experienced with three distinct seasons, summer (March-June), rainy (July-October), and winter (November–February).

### Study design

We designed this retrospective cohort study on the selected villages of the Raghopur block to assess the effect of long term exposure of groundwater arsenic pollution of the people and associated risk of PKDL development. All PKDL patients reporting to Rajendra Memorial Research Institute of Medical Sciences center for treatment during 2009–2014 from the Raghopur block in Vaishali district, were included in this study. After collecting information from the electronic center database, trained physicians (unaware of the arsenic data) interviewed the PKDL patients in person in their village and all relevant data were collected retrospectively in a questionnaire format. The information about treatment regimens during VL was cross-checked with the existing center database by interviewing the patients and/or their close relatives. Furthermore, detailed data about their treatment outcome during VL and PKDL, were recorded. The data included information on treatment failure, treatment success, relapse or death. Relevant covariate data was derived from the center database and the baseline interviews. The socio-demographic data included age in years, sex and fundamental education (in years). The height, weight, basal metabolic rate, systolic blood pressure etc data were collected from the center database. Besides socio-demographic, epidemiological and clinical data, we also collected data about their drinking and other utility water sources, and information about the amount of consumption of cooked rice in the family.

### Assessment of arsenic exposure of the PKDL patients

All probable water sources were identified around the patient’s house and water was collected for evaluation of arsenic concentration. The protocol for arsenic evaluation in hand pumps, using field test kits (FTK), had already been approved by Government of Bihar, Government of Uttar Pradesh, and UNICEF, for detection of arsenic over large areas. Notably, the initial assessment of ground water arsenic contamination was performed by Field Test Kits (FTK) designed by National Chemical Laboratory, Pune. Next, all test samples for arsenic results were retested using flow-injection hydride-generation atomic absorption spectrometry (FI-HG-AAS) at the Department of Environment and Water Management, A.N. College, Patna. This was followed by recording of the locations of arsenic-affected hand pumps or water bodies, using Global Positioning System (GPS) units, was done, followed by mapping of the arsenic occurrences. The other references used were block maps and topographical maps. Arsenic dose (μg/day) was calculated as: (arsenic concentration in water of the primary source, μg/L) X (self-reported daily amount of water from that source, L per day), n = 139. Simultaneously, the total arsenic concentration in urine was divided by the concentration of creatinine in the urine to achieve a creatinine-adjusted total arsenic concentration in the urine expressed as μg/g creatinine; as described earlier [31]. For that, total arsenic concentration in urine was assessed by FI-HG-AAS with a detection limit of 2.0 μg/L. Creatinine level in the urine was measured with a commercial colorimetric kit [Sigma Aldrich, USA]. Total arsenic concentration in urine and arsenic dose per day was quartiled according to the baseline distribution of the cohort. We also involved dermatological examination of randomly selected individuals living in the study cohort to assess the effect of chronic arsenic exposure of the population. The presence or absence of arsenic-induced skin symptoms, including melanosis, suspicious spotty depigmentation / pigmentation over trunk /limbs, diffuse thickening of soles and palms, pigmentation involving the undersurface of tongue and/or buccal mucosa, leucomelanosis or keratosis, was examined by a clinician with ample experience in diagnosing arsenicosis cases and was blinded to the exposure level.

### GIS layers preparation and feature identification

For the study area, survey of India’s (SOI) toposheet number 72J of the scale 1:50,000 was used for preparation of the base map. The topographic map was geo-referenced with the latitudes and longitudes using the ArcGIS software v9.3 (ESRI, Redlands, CA, USA) to demarcate the boundary of study villages and interpret the remote sensing data. For delineation of spatial pattern of the river/surface waterlogged areas as well as land uses/land covers of the study villages, screen shot of high resolution satellite imageries were downloaded from Google Earth desktop version-6.2 (USDA Farm Service Agency, 2013 Digital Globe). The raw satellite imageries were georeferenced, mosaicked and analyzed using the ERDAS imagines software v9.2 (Hexagon Geospatial, USA; formerly ERDAS, Inc.). Features classes were identified based on the visual image interpretation elements (such as tone, texture, shape, pattern and association) and results were verified by ground truth collection of earth phenomenon using Global Positioning System (GPS). Online image digitization and overlay technique was used to create the feature layers.

### Geo-database development of PKDL cases and arsenic contamination in ground water

A GIS based village level digital database of PKDL cases report and arsenic contamination in ground water was developed. Village wise cases data were short listed using the address given in the registers. All the patient locations were verified in situ by GPS device to geo-coding the case data on the map. Individual point location of PKDL cases were plotted on the arsenic distribution map. Spatial distribution of polygonal inhabitant areas were marked and overlaid on integrated GIS map. Finally, GIS integrated village boundary layer was used to store the quantitative values for mapping, visualization, statistical analysis and represent the results.

### Statistical analyses

A stepwise multivariate logistic regression model was used to assess the association of chronic arsenic exposure and risk of PKDL development in treated VL patients. Data on variables were based on prior casual knowledge and was derived from the baseline interview forms. This model was accountable to possible confounding. Each covariate was individually analyzed for the association by logistic regression. The model was primarily adjusted for age (in years) and gender. Later, the model was further adjusted in multivariate analysis for presence of potential confounders viz. body-mass index (BMI in kg/m^2^), systolic blood pressure (mm Hg), caste status, fundamental education (in years), previous VL treatment with SSG in the family, place of treatment during VL and time to treatment (in years). Confounders with missing or incomplete data were excluded from the analysis. As several participants used the same well or hand pump for water, we included clustering in our analysis using SEs for the hazard model. Odds ratios (ORs) were estimated and their 95% CIs were evaluated. All statistical analyses were carried out with SAS 8.2 software (SAS Institute Inc., USA).

## Results

### Spatial distribution of the study cohort

The study cohort Raghopur block is located in the southern part of Vaishali district. Using online image digitalization and overlay techniques, environmental features of the cohort were classified into three categories such as river/surface water bodies, settlement/built-up areas (inhabitants places) and other land use/land covers (mixed of agricultural crops, sparse vegetation and grass land) (Fig 1). The seven study villages of the cohort were distributed in a geographic area of approximately 11362.66 hectare. River stream areas covered 1.65% (187.72 hectare) of land and were distributed evenly throughout the seven villages. One hundred thirteen settlement clusters (as per satellite data) were identified from all seven villages where the population was at risk for kala-azar. Area of these settlement clusters ranged from 0.155 to 137.21 hectare. Spatial distribution of settlement cluster shows a significant interconnection (p<0.001) amongst the study villages.

### Study population

**Fig 2:**
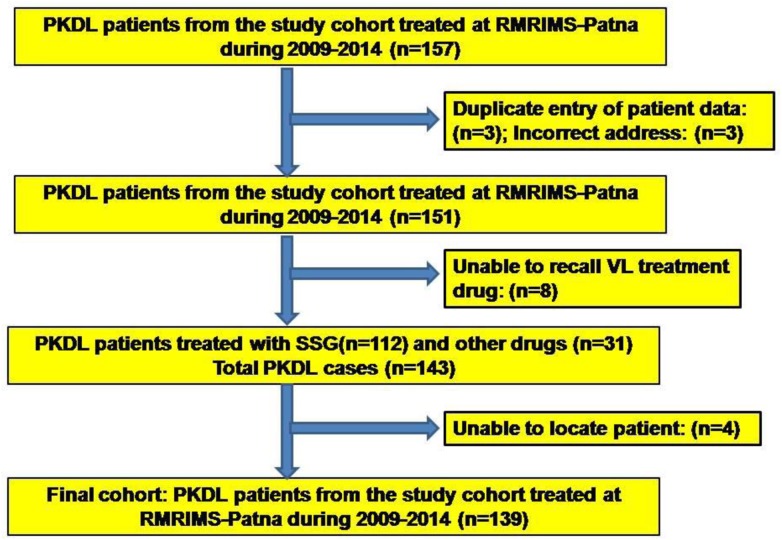
Flow chart of the study population. Flow chart of the study population from PKDL patients who were treated at Rajendra Memorial Research Institute of Medical Sciences (RMRIMS), Patna, Bihar between 2009 and 2014.

We hypothesized that the long term exposure to groundwater arsenic contamination is an additional risk factor for development of PKDL in patients treated for VL. For that, we identified 157 PKDL patients from the study area (Fig 1), who were treated at Rajendra Memorial Research Institute of Medical Sciences (RMRIMS), Patna, Bihar during 2009–2014 (Fig 2). Among them, some were excluded from the study as either history of treatment during VL were not found for eleven patients (n = 11) or were duplicate entries (n = 3). Therefore, finally, one hundred and forty three (n = 143) PKDL patients were included in the study. After receiving their treatment data during VL episode, we divided them into two subgroups: Group A- patients treated with SSG during VL (n = 112) and Group B- patients treated with other drugs (eg. Amphotericin B, Miltefosine, Paramomycin, Ambisome etc.) during VL episode (n = 31) (Fig 2). After collecting informations from the center database, the PKDL patients were visited in their village and all relevant data were collected retrospectively by clinicians in a questionnaire format. Among the 143 PKDL patients, 92 (64.3%) were available for the interviews. Thirty-five (24.4%) subjects were living outside the study area during the visit due to migration to other cities for jobs, three subjects could not be located due to misinformation in address and one subject was dead. The relatives of the rest twelve subject were interviewed to gather information. Based on the information, total thirty one (21.6%) patients were not treated with SSG, i.e treated with Amphotericin B (n = 13), Miltefosine (n = 7), Paramomycin (n = 8), Ambisome (n = 3) etc., were also included in the study. Finally, a cohort of one hundred and thirty nine (n = 139) subjects were found as the study population in Raghopur block (Fig 2).

### The PKDL cases

**Fig 3:**
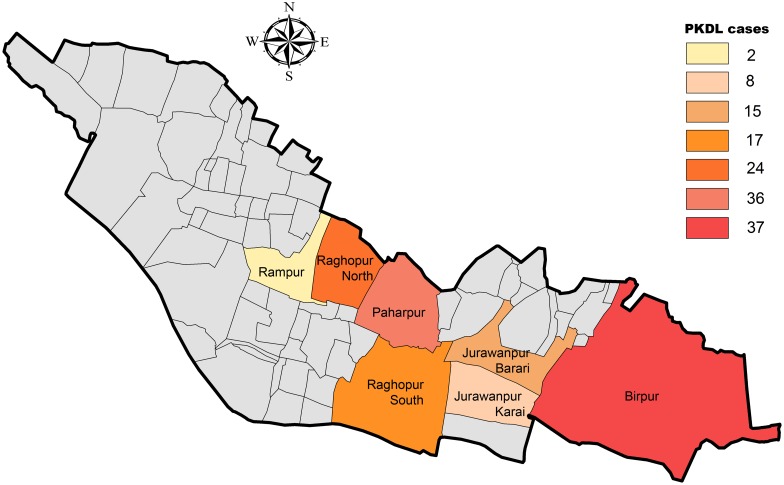
Village-wise PKDL case distribution. Village-wise PKDL case distribution in the study cohort of Raghopur block, Vaishali district, Bihar, India.

The cohort of 139 patients was aged between 5 to 64 years with males to females 3:2. The comparative status for history of VL episodeof all PKDL cases in the cohort is shown in Table 1. No significant difference was observed between the history of VL episode between the two groups of PKDL patients in the study cohort. (Table 1). The characteristics of confounders of all PKDL cases are shown in Table 2. The village-wise distribution of PKDL cases is shown in Fig 3. Most of the patients (92%) were living in this block for the last 10.8 to 26.4 years. All patients were recruited during 2009–2014 from the outpatient/inpatient departments of Rajendra Memorial Research Institute of Medical Sciences (ICMR), Patna, India. All clinical investigations were ethically approved and performed as per the Declaration of Helsinki. The PKDL patients reported with macular/nodular/mixed polymorphic (macules with presence of nodules and/or papules) non-anesthetic lesions on face/forelimbs/shoulder. The previous history of VL and treatment used during the VL episode was recorded along with other information. These suspected PKDL cases were diagnostically confirmed by rK39 strip test followed by LD body detection in Giemsa-stained lesion-biopsy specimens. For smear negative samples (especially in macular PKDL lesions), DNA was isolated from skin lesion samples and PCR was conducted to detect kDNA of the parasite for confirmation of PKDL, as described earlier [32]. All PKDL patients were treated with Amphoterin B (AmB; 1 mg/kg body weight) as alternate-day infusions for 40 days for 5 months with two 15-day breaks between the courses.

**Table 1:** Comparative status for history of VL episode of the PKDL cases in the study cohort.

Study groups in the cohort	Group A: PKDL patients treated with SSG during VL episode (n = 108)	Group B: PKDL patients treated with other drugs during VL episode (n = 31)
**Previous history of VL in the family**		
No	97 (89.8%)	26 (83.8%)
Yes	10 (9.2%)	4 (12.9%)
**Time between VL episode and PKDL, years**		
0–5	95 (87.9%)	25 (80.6%)
>5	13 (12.03%)	6 (19.3%)
**Completed drug treatment schedule during VL episode**		
No	37 (34.2%)	9 (29.03%)
Yes	71 (65.7%)	22 (70.9%)
**Treatment status after VL episode**		
Failure	28 (25.9%)	5 (16.1%)
Success	80 (74.07%)	26 (83.8%)
**Place from where VL treatment was received**		
Private clinic	11 (10.1%)	3 (9.6%)
Government center	97 (89.8%)	28 (90.3%)

**Table 2:** Selected confounders in the cohort.

	Arsenic concentration (μg/mL) in water sources	Baseline cohort (n = 139)
**Sex**		
Male	117.2 (45.1)	108 (77.6%)
Female	109.4 (39.6)	31 (22.3%)
**Age (yrs)**		
5–15	104.2 (22.3)	27 (19.4%)
16–30	121.4 (36.3)	59 (42.4%)
31–50	110.6 (39.4)	39 (28.05%)
51–70	107.1 (34.6)	14 (10.07%)
**Body mass index (kg/m**^**2**^**)**		
<18.5	120.7 (31.6)	73 (52.5%)
18.5–24.9	102.6 (28.9)	44 (31.6%)
≥25	99.4 (22.8)	22 (15.8%)
**Systolic blood pressure (mmHg)**		
<140	109.3 (14.2)	112 (80.5%)
≥140	98.7 (22.5)	27 (19.4%)
**Fundamental Education (yrs)**		
0	112.4 (24.7)	45 (32.3%)
1–10	101.7 (18.6)	84 (60.4%)
11–15	91.6 (24.4)	10 (7.1%)
**Caste status**		
Higher	84.6 (32.5)	31 (22.3%)
Backward	110.1 (24.6)	108 (77.6%)

### Mapping, visualization of arsenic contamination in groundwater and PKDL occurrences in the study cohort

In this study, the spatial distribution of the arsenic contaminated areas was studied by GIS based mapping techniques. GIS based thematic map showed the spatial distribution of arsenic contaminated villages in the focused study cohort at Raghopur block (Fig 3). The GIS map indicates low, medium and high spatial variability in arsenic concentration through the color scale (Fig 3). Spatial distribution of polygonal inhabitant areas were marked and overlaid on integrated GIS map.

**Fig 4:**
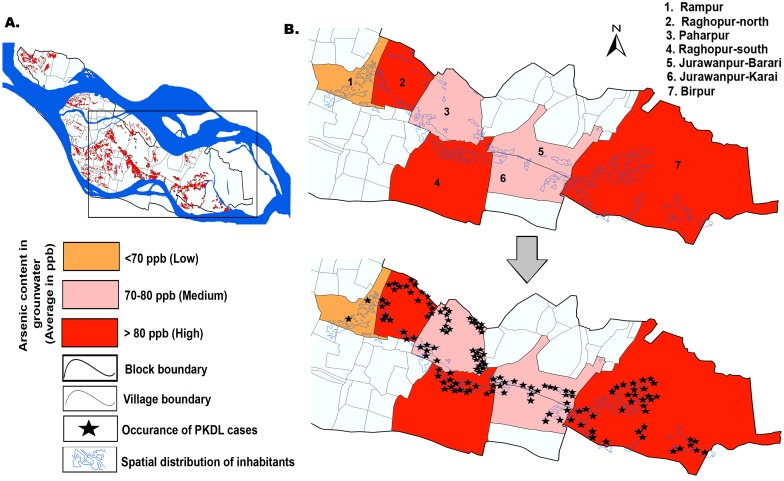
Distribution of arsenic contamination in groundwater and PKDL cases in the study cohort. A: Map of Raghopur block showing spatial distribution of inhabitants, B. Distribution of arsenic in groundwater in seven villages of the study cohort, C. Overlap of location of PKDL cases with distribution of arsenic in groundwater in seven villages of the study cohort.

After plotting individual geocoded cohort addresses of PKDL cases on the arsenic distribution map using spatial join functionality, we found that 7 villages in Raghopur block of Vaishali district in Bihar indicate that arsenic concentrations are high in groundwater collected from the youngest alluvial terraces (Fig 4). Out of the 437 samples tested, 409 samples were confirmed to have arsenic content exceeding the World Health Organization (WHO) guideline value (10μg/L) of arsenic. Most samples (92%) tested were having more than 50 ppb. of arsenic content. The wells were considerably less contaminated than the hand pumps in the block. Eighty four (60.4%) PKDL patients had at least one highly arsenic contaminated hand pump in their close vicinity and reported of using the water for drinking and other requirements. However, only 12 (8.3%) PKDL patient were aware of arsenic contamination in their drinking water.

A positive co-relation was established between PKDL cases incidence and the level of arsenic concentration in the ground water. Results denoted that the highest arsenic content is 432 ppb. in a hand pump, collected from Birpur village, east of Raghopur block. This place was located very near to the coast of Ganges. Interestingly, 37 (26.6%) PKDL patients reported from Birpur village of the Raghopur block during the study period (Figs 3 and 4). Simultaneously, in Raghopur-north village, the highest arsenic level detected is 375 ppb. In a hand pump located near the house of a PKDL patient. Next, the highest arsenic value of 321 ppb. was recorded from Ragopur-south village, Raghopur block (Fig 4A and 4B). Notably, forty one (29.4%) PKDL cases reported from both Raghopur (south and north) villages.

Contrastingly, the arsenic load of Paharpur village was medium (70–80 ppb) (Fig 4A and 4B). The highest arsenic level detected was 76 ppb. near the Raghopur-Diyara island area, followed by 72 ppb. in Paharpur. However, in Jurawanpur Barari and Jurawanpur Karai village, the highest arsenic levels read were 74 ppb. and 78 ppb. respectively (Figs 3 and 4). Both these areas were comparatively less populated in the block. The lowest levels of arsenic content in Rampur village was within 70 ppb. (Fig 4), making it the least arsenic contaminated village surveyed in the Raghopur block. Interestingly, only two PKDL patient reported from this area.

### Arsenic exposure

**Fig 5:**
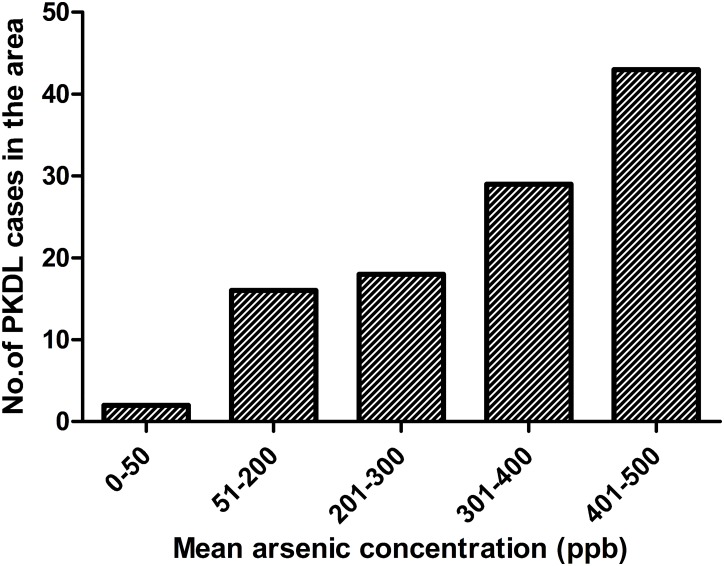
Distribution of PKDL cases in respect to variable arsenic concentrations in the study cohort.

Table 2 shows the distribution of socio-demographic, clinical and exposure characteristics of the PKDL patients in the baseline study cohort. We also used data on potential confounding factors for adjustment of the cohort data. After adjustment for potential confounding, the estimated summary attributable proportion based on arsenic concentration in water for PKDL risk in treated VL patients was 35%. We investigated whether arsenic load has relation with number of cases reported from the village. Our results also denoted that number of cases increased with arsenic load, i.e. more PKDL cases were reported from highly arsenic contaminated areas (Figs 4 and 5 and Table 3).

**Table 3:** Odds ratios (ORs) for risk of PKDL development in the study participants in association to arsenic exposure.

	PKDL development (n = 139) *		
	OR (95% CI)	Z statistics	P value
**Arsenic concentration (μg/L) in the water source**			
0.1–10.0	1.0		
10.1–200.0	1.85 (1.13–3.03)	2.46	0.01
200.1–432.0	2.31 (1.39–3.8)	3.2	0.001
**Arsenic dose (μg per day)**			
0.05–50.0	1.0		
50.1–247.0	1.32 (0.77–2.25)	1.03	0.2
247.1–1076.0	1.21 (0.71–2.03)	0.72	0.4
**Total arsenic content in urine (μg/g)**			
5.0–123.0	1.0		
123.1–436.0	1.35 (0.80–2.28)	1.13	0.25
436.1–1278.0	1.40 (0.87–2.25)	1.4	0.16
1278.1–4000.0	1.61 (0.99–2.6)	1.9	0.05

*Multivariate analysis were adjusted for confounders body-mass index (BMI in kg/m^2^), systolic blood pressure (mm Hg), caste status, fundamental education (in years), previous VL treatment with SSG or any other drug in the family etc.

Arsenic exposure (in terms of baseline arsenic concentration in the water sources, arsenic dose per day, and total arsenic concentration in the urine samples) was well associated with the risk of PKDL development (Table 3). Interestingly, the risk of PKDL development increased with arsenic dose per day and arsenic levels found in the urine samples (Table 3). The range of Pearson correlation coefficients for the level of arsenic exposures were 0.72–0.94, the most strongest factors were arsenic concentration in the water source and arsenic dose per day of the individual for PKDL development. With respect to the data of the ordinal exposure, a one-quartile increase in arsenic concentration of water source or arsenic intake per day or arsenic level in the urine, there was about 12–16% increase in PKDL risk in this multivariate model (Table 3). The multivariate-adjusted OR for comparison of high baseline arsenic exposure to low baseline arsenic exposure of the individuals in the study cohort was 1.66 (95% CI: 1.02–2.7; p = 0.04) and demonstrated a positive role of arsenic exposure on PKDL development.

We also involved dermatological examination of randomly selected individuals living in the study cohort to assess the effect of chronic arsenic exposure of the population. The presence of arsenic-induced skin symptoms like melanosis, spotty depigmentation/ pigmentation over trunk /limbs, diffuse thickening of soles and palms, pigmentation on or below tongue or keratosis, was noted. Severe symptoms like diffuse verrucous lesions of the soles with cracks and fissures were noted in two individuals.

## Discussion

The findings of our current study suggest significant effect of arsenic exposure through groundwater contamination on risk of PKDL development in VL patients, treated with SSG or other drugs during the VL episode. As contained in water, daily intake of arsenic creates a medical condition by elevated levels of sodium arsenite in different parts of the body. Recently, Perry et al. demonstrated that long-term arsenic exposure and subsequent adaptation of *L*.*donovani* to sublethal levels of arsenic in their human hosts may have led to cross-resistance to antimonials and the arsenic hotspots in Bihar coincided with areas with SSG treatment failure [28, 33, 34]. The dermal effects following the exposure to arsenic are hallmarks of arsenic toxicity, where hyperkeratosis and hyperpigmentation are the commonest examples [14, 35]. Evidences also suggest that SSG directly or indirectly influences the incidence of PKDL [30]. However, it is important to note that about 27% of PKDL also develops in VL patients treated with other drugs, like amphotericin B, ambisome, miltefosine, miltefosine—amphotericin B, or paromomycin in Bihar [30, 36, 37]. It is plausible that greater number of PKDL cases from SSG-treated VL cases can also be due to massive use of SSG in Bihar during the 80s, when no other option was readily available for VL treatment in this area. Therefore, besides the risk factor of ineffective treatment of SSG, the environmental factors prevailing in the area may play additional risk for PKDL development and further studies are required to strengthen this point.

In the study cohort, all probable water sources were identified around the patient’s house and high levels of arsenic concentration was found. Interestingly, human exposure to inorganic arsenic is associated with an increased risk of dermal malignancies and acts as a cofactor in the development of skin tumors in combination with ultraviolet (UV) irradiation [38]. Furthermore, arsenic hazard studies report close link between the clothing habits of individuals and health risk development; potentiating the dermatological effect of arsenic in the presence of UV-ray exposure through sunlight [39, 40]. Interestingly, PKDL presents with a spectra of dermal manifestations, especially in the sun-exposed areas of the body, relating lesional patterns with the clothing habits of individuals and with exposure to UV radiation [30, 41, 42]. Sun exposure has been reported to induce rapid immunological changes in skin and peripheral blood [43], and also to help in immunosupression through reduction in dermal DC subset populations in psoriasis patients [44]. The VL patients, residing in the study cohort, are vulnerable to both arsenic exposure and to over-exposure to sunlight as their main occupation is farming. Another study reports that occupational exposure of arsenic among workers in a glass plant, with blood arsenic levels five times higher compared to the control group, leads to increased DNA damage in leukocytes [45]. As leukocytes play a major role in cure of VL, over-exposure of arsenic may restrict leishmanicidal functions of leukocytes in VL patients during treatment, leading to escape of parasites from the killing mechanism. Therefore, arsenic groundwater contamination may act as an additive risk factor for PKDL development in Bihar.

Interestingly, reactive oxygen species (ROS)-mediated oxidative damage is a common denominator in arsenic pathogenesis [46]. In addition, arsenic also induces severe morphological changes in mitochondrial integrity leading to oxidant-induced DNA damage [46]. Free radical formation from the superoxide radical, combined with glutathione-depleting agents, increase the sensitivity of cells to arsenic toxicity [46]. Therefore, individuals exposed to arsenic, have an increased formation of ROS/RNS, including superoxide radical, singlet oxygen, hydroxyl radical (OH•) and hydrogen peroxide. The parasites of VL generally reside in the liver and spleen tissue microenvironments and influence qualitative and quantitative aspects of the host immunity [47]. Oxidative and nitrosative stress components have serious adverse effects on the host during VL and PKDL infection. Furthermore, non-restoration of normal activities of peroxisomal catalase and superoxide dismutase in the host has been found responsible for unsuccessful clearance of *Leishmania* parasites from liver and spleen [48]. The prolonged exposure to groundwater arsenic contamination probably adds up to the increased oxidative stress and peroxisomal dysfunction in the host. It can be suggested that long time arsenic exposure may exert its influence on keratinocytes and lymphocytes, leading to modulation of cytokines that may promote development of PKDL in treated VL patients, which may also have influence on the incidence of VL in Bihar.

The findings of this study suggested a positive co-relation between the incidence of PKDL cases and the level of arsenic concentration in the ground water of the cohort. However, it is difficult to interpret the exact role of arsenic on number of PKDL cases reporting from a contaminated area as background incidence of VL may have some impact. For example, the low number of PKDL cases reporting from Rampur village could also be due to low VL incidence in that area. Our findings also suggested that there were no apparent differences between history of VL episode between the two groups of PKDL cases in the cohort (Table 1). This also indicates a probable role of environmental factors, like arsenic exposure, in PKDL development. Interestingly, the immune system has been reported to be a sensitive target for arsenic exposures that may be associated with decreased host resistance to infectious agents [49, 50]. Arsenic causes significant changes in T-cell secreted cytokine levels with altered T-cell activation status leading to immunosuppression favoring opportunistic infections in exposed individuals. Notably, arsenite also suppresses the activation of Th1 (T bet) cells, and alters the percentages of Th17 (RORγt) and T-reg (FoxP3) population [51]. As T-cells are crucial deciders for the fate of VL infection, chronic arsenic exposure could also have contributed for incidence of VL. Notably, exposure to arsenic is associated with an increased prevalence of malnutrition [52], leading to susceptibility to skin lesions [53]. Malnutrition also induces immunosuppression. There is prevalence of mass malnutrition among VL patients and their family members in Bihar. Therefore, it is plausible that long term arsenic exposure could also have contributed for incidence of VL in the affected areas, finally also co-affecting PKDL incidence. However, no definite conclusions can be drawn without further studies on this aspect.

Reportedly, PKDL-causing *Leishmania donovani* strains express higher levels of certain surface proteins that are associated with dermatotropism of the parasite [54]. Besides possible effect of ineffective treatment of VL or other risk factors, exposure to arsenic may additionally contribute to the emergence of PKDL in Bihar, through its pro-dermatotropic effects on the parasite surface. Further work is underway to study the parasite protein expression profile during chronic arsenic exposure that would further enlighten the mechanism of parasite dermatotropism. The current work highlights the need to consider environmental factors like arsenic exposure as an additional risk factor for PKDL development in India. However, the role of arsenic exposure on occurrence of VL cannot be ruled out. Further extensive mechanistic and epidemiological studies are required to assess the real role of arsenic exposure on PKDL development.

## Supporting Information

S1 Checklist STROBE CHECKLIST. (DOCX)
