# Supplementary material for: Chronic Arsenic Exposure and Risk of Post Kala-azar Dermal Leishmaniasis Development in India: A Retrospective Cohort Study
Source: PLoS Negl Trop Dis. 2016 Oct 24;10(10):e0005060. doi: 10.1371/journal.pntd.0005060 (PMC5077161; doi:10.1371/journal.pntd.0005060)
Supplement: S1 Checklist — (DOCX) [file pntd.0005060.s001.docx]

**STROBE Statement—Checklist of items that should be included in reports of cohort studies**

|  | Item No | Recommendation | Page number | Relevant text from manuscript |
| --- | --- | --- | --- | --- |
| **Title and abstract** | 1 | (a) Indicate the study’s design with a commonly used term in the title or the abstract | 1 | “A retrospective cohort study” |
|  |  | (b) Provide in the abstract an informative and balanced summary of what was done and what was found | 2 | Abstract |
| Introduction | | | | |
| Background/rationale | 2 | Explain the scientific background and rationale for the investigation being reported | 5,6 | Arsenic poisoning is a medical condition caused by elevated levels of sodium [arsenite](http://en.wikipedia.org/wiki/Arsenic) in different parts of the body. Notably, the problem of groundwater contamination in 18 out of the 38 districts in Bihar has reached alarming proportions since arsenic in groundwater was first reported in June 2002 in Bhojpur district [22-24].  Therefore, we hypothesized of the contributing role for some environmental factors, like chronic exposure of arsenic contaminated groundwater, in development of PKDL. In this study, in light of the risk of CAT-associated dermal manifestations, we hypothesized that the long term exposure to groundwater arsenic contamination acts as a risk factor for development of PKDL in patients treated for VL. |
| Objectives | 3 | State specific objectives, including any prespecified hypotheses | 6 | Therefore, we hypothesized of the contributing role for some environmental factors, like chronic exposure of arsenic contaminated groundwater, in development of PKDL. In this study, in light of the risk of CAT-associated dermal manifestations, we hypothesized that the long term exposure to groundwater arsenic contamination acts as a risk factor for development of PKDL in patients treated for VL. |
| Methods | | | | |
| Study design | 4 | Present key elements of study design early in the paper | 7,8 | “Retrospective cohort study”, “questionnaire”, “measurement of arsenic concentration in water sources as a proxy for arsenic contamination”, “measurement of arsenic concentration in urine as a proxy for arsenic exposure”, “GIS based village level digital database of PKDL cases report and arsenic contamination in ground water” |
| Setting | 5 | Describe the setting, locations, and relevant dates, including periods of recruitment, exposure, follow-up, and data collection | 7 | “Raghopur block of the Vaishali District ”, “2009-2014”, “questionnaire” “Institutional Clinical records, baseline interviews |
| Participants | 6 | (a) Cohort study—Give the eligibility criteria, and the sources and methods of selection of participants. Describe methods of follow-up:  Case-control study—Give the eligibility criteria, and the sources and methods of case ascertainment and control selection. Give the rationale for the choice of cases and controls  Cross-sectional study—Give the eligibility criteria, and the sources and methods of selection of participants.  (b) Cohort study—For matched studies, give matching criteria and number of exposed and unexposed  Case-control study—For matched studies, give matching criteria and the number of controls per case | 8 | All PKDL patients reporting to Rajendra Memorial Research Institute of Medical Sciences centre for treatment during 2009-2014 from the Raghopur block in Vaishali district, were included in this study.  The information about treatment regimes during VL was cross-checked with the existing centre database by interviewing the patients and/or their close relatives. Furthermore, detailed data about their treatment outcome during VL and PKDL, were recorded. |
| Variables | 7 | Clearly define all outcomes, exposures, predictors, potential confounders, and effect modifiers. Give diagnostic criteria, if applicable | 8,9  13, 14 | Being totally intercepted by the Ganges, we selected Raghopur block of the Vaishali District, Bihar, as the target cohort for our retrospective study (Figure 1).  Relevant covariate data was derived from the centre database and the baseline interviews. The sociodemographic data included age in years, sex and fundamental education (in years). The height, weight, basal metabolic rate, systolic blood pressure etc data were collected from the center database. Besides sociodemographic, epidemiological and clinical data, we also collected data about their drinking and other utility water sources, and information about the amount of consumption of cooked rice in the family.  The presence or absence of arsenic-induced skin symptoms, ……was examined by a clinician with ample experience in diagnosing arsenicosis …… to the exposure level.  These suspected PKDL cases were diagnostically confirmed by rK39 strip test followed by LD body detection in Giemsa-stained lesion-biopsy specimens. For smear negative samples (especially in macular PKDL lesions), DNA was isolated from skin lesion samples and PCR was conducted to detect kDNA of the parasite for confirmation of PKDL. |
| Data sources/ measurement | 8* | For each variable of interest, give sources of data and details of methods of assessment (measurement). Describe comparability of assessment methods if there is more than one group | 9 | All probable water sources were identified around the patient’s house and water was collected for evaluation of arsenic concentration.  This was followed by recording of the locations of Arsenic-affected hand pumps or water bodies, using Global Positioning System (GPS) units, was done, followed by mapping of the arsenic occurrences.  All test samples for arsenic results were retested using flow injection hydride-generation atomic absorption spectrometry (FI-HG-AAS). This was followed by recording of the locations of Arsenic-affected hand pumps or water bodies, using Global Positioning System (GPS) units, was done, followed by mapping of the arsenic occurrences.  Arsenic concentration in urine was assessed by FI-HG-AAS with a detection limit of 2.0 µg/L. Creatinine level in the urine was measured with a commercial colorimetric kit. |
| Bias | 9 | Describe any efforts to address potential sources of bias | 8,9 | After collecting informations from the electronic centre database, trained physicians (unaware of the arsenic data) interviewed the PKDL patients in person in their village and all relevant data were collected retrospectively in a questionnaire format. The information about treatment regimes during VL was cross-checked with the existing centre database by interviewing the patients and/or their close relatives.  The presence or absence of arsenic-induced skin symptoms, ………, was examined by a clinician with ample experience in diagnosing arsenicosis cases and was blinded to the exposure level. |
| Study size | 10 | Explain how the study size was arrived at | 8 | All PKDL patients reporting to Rajendra Memorial Research Institute of Medical Sciences centre for treatment during 2009-2014 from the Raghopur block in Vaishali district, were included in this study. |
| Quantitative variables | 11 | Explain how quantitative variables were handled in the analyses. If applicable, describe which groupings were chosen and why | 8, 9,10 | The data included informations on treatment failure, treatment success, relapse or death. Relevant covariate data was derived from the centre database and the baseline interviews. The sociodemographic data included age in years, sex and fundamental education (in years). The height, weight, basal metabolic rate, systolic blood pressure etc data were collected from the center database. Besides sociodemographic, epidemiological and clinical data, we also collected data about their drinking and other utility water sources, and information about the amount of consumption of cooked rice in the family.  Arsenic dose (µg/day) was calculated as: (arsenic concentration in water of the primary source , µg/L) X (self-reported daily amount of water from that source, L per day), n=139. Simultaneously, the total arsenic concentration in urine was divided by the concentration of creatinine in the urine to achieve a creatinine-adjusted total arsenic concentration in the urine expressed as µg/g creatinine; as described earlier [31].For that, total arsenic concentration in urine was assessed by FI-HG-AAS with a detection limit of 2.0 µg/L. Creatinine level in the urine was measured with a commercial colorimetric kit [Sigma Aldrich, MO, USA]. Total arsenic concentration in urine and arsenic dose per day was quartiled according to the baseline distribution of the cohort. |
| Statistical methods | 12 | (a) Describe all statistical methods, including those used to control for confounding | 10,11 | Described in the paragraph under heading “Statistical analysis” |
|  |  | (b) Describe any methods used to examine subgroups and interactions | N/A |  |
|  |  | (c) Explain how missing data were addressed | 11 | Described in the paragraph under heading “Methods” |
|  |  | (d) Cohort study—If applicable, explain how loss to follow-up was addressed  Case-control study—If applicable, explain how matching of cases and controls was addressed  Cross-sectional study—If applicable, describe analytical methods taking account of sampling strategy | N/A |  |
|  |  | (e) Describe any sensitivity analyses |  |  |
| Results | | | | |
| Participants | 13* | (a) Report numbers of individuals at each stage of study—eg numbers potentially eligible, examined for eligibility, confirmed eligible, included in the study, completing follow-up, and analysed. | 12, 13 | For that, we identified 157 PKDL patients from the study area (Figure 1), who were treated at Rajendra Memorial Research Institute of Medical Sciences (RMRIMS), Patna, Bihar during 2009-2014 (Figure 2). Finally, a cohort of one hundred and thirty nine (n=139) subjects were found as the study population in Raghopur block (Figure 2), |
|  |  |  |  | Among the 143 PKDL patients, 92 (64.3%) were available for the interviews. Thirty-five (24.4%) subjects were living outside the study area during the visit due to migration to other cities for jobs, three subjects could not be located due to misinformation in address and one subject was dead. The relatives of the rest twelve subject were interviewed to gather informations. Based on the informations, total thirty one (21.6%) patients were not treated with SSG, i.e treated with Amphotericin B (n=13), Miltefosine (n=7), Paramomycin (n=8), Ambisome(n=3) etc., were also included in the study. Finally, a cohort of one hundred and thirty nine (n=139) subjects were found as the study population in Raghopur block (Figure 2), |
|  |  | (b) Give reasons for non-participation at each stage | N/A |  |
|  |  | (c) Consider use of a flow diagram | Figure 2 |  |
| Descriptive data | 14* | (a) Give characteristics of study participants (eg demographic, clinical, social) and information on exposures and potential confounders | 8.14.16 | Under headings “Study design” and “Arsenic exposure”  Also in Table 2 |
|  |  | (b) Indicate number of participants with missing data for each variable of interest | 12 | Among the 143 PKDL patients, 92 (64.3%) were available for the interviews. Thirty-five (24.4%) subjects were living outside the study area during the visit due to migration to other cities for jobs, three subjects could not be located due to misinformation in address and one subject was dead. |
|  |  | (c) Summarise follow-up time (eg, average and total amount) | Not stated | Retrospective study, the endpoints are different |
| Outcome data | 15* | Report numbers of outcome events or summary measures over time | 13,14,17 | Specified in Tables 1, 2 and 3 |
| Main results | 16 | (a) Give unadjusted estimates and, if applicable, confounder-adjusted estimates and their precision (eg, 95% confidence interval). Make clear which confounders were adjusted for and why they were included | 10,11, 16-18, | Mentioned under headings ‘Statistical analysis’, Arsenic exposure’, and in Table 3. |
|  |  | (b) Report category boundaries when continuous variables were categorized | 17 | Table 3 |
|  |  | (c) If relevant, consider translating estimates of relative risk into absolute risk for a meaningful time period | N/A |  |
| Other analyses | 17 | Report other analyses done—eg analyses of subgroups and interactions, and sensitivity analyses | N/A |  |
| Discussion | | | | |
| Key results | 18 | Summarise key results with reference to study objectives | 20, 21 | The findings of this study suggested a positive co-relation between the incidence of PKDL cases and the level of arsenic concentration in the ground water of the cohort.  The current work highlights the need to consider environmental factors like arsenic exposure as an additional risk factor for PKDL development in India. However, the role of arsenic exposure on occurrence of VL cannot be ruled out. Further extensive mechanistic and epidemiological studies are required to assess the real role of arsenic exposure on PKDL development. |
| Limitations | 19 | Discuss limitations of the study, taking into account sources of potential bias or imprecision. Discuss both direction and magnitude of any potential bias. | 20,21 | Retrospective nature of the study.  The findings of this study suggested a positive co-relation between the incidence of PKDL cases and the level of arsenic concentration in the ground water of the cohort. However, it is difficult to interpret the exact role of arsenic on number of PKDL cases reporting from a contaminated area as background incidence of VL may have some impact.  There is prevalence of mass malnutrition among VL patients and their family members in Bihar. Therefore, it is plausible that long term arsenic exposure could also have contributed for incidence of VL in the affected areas, finally also co-affecting PKDL incidence. However, no definite conclusions can be drawn without further studies on this aspect.  Besides possible effect of ineffective treatment of VL or other risk factors, exposure to arsenic may additionally contribute to the emergence of PKDL in Bihar, through its pro-dermatotropic effects on the parasite surface. Further work is underway to study the parasite protein expression profile during chronic arsenic exposure that would further enlighten the mechanism of parasite dermatotropism. |
| Interpretation | 20 | Give a cautious overall interpretation of results considering objectives, limitations, multiplicity of analyses, results from similar studies, and other relevant evidence | 19,20,21 | Interestingly, human exposure to inorganic arsenic is associated with an increased risk of dermal malignancies and acts as a cofactor in the development of skin tumours in combination with ultraviolet (UV) irradiation [38]. Furthermore, arsenic hazard studies report close link between the clothing habits of individuals and health risk development; potentiating the dermatological effect of arsenic in the presence of UV-ray exposure through sunlight [39, 40]. Interestingly, PKDL presents with a spectra of dermal manifestations, especially in the sun-exposed areas of the body, relating lesional patterns with the clothing habits of individuals and with exposure to UV radiation [30, 41, 42]. Sun exposure has been reported to induce rapid immunological changes in skin and peripheral blood [43], and also to help in immunosupression through reduction in dermal DC subset populations in psoriasis patients [44]. The VL patients, residing in the study cohort, are vulnerable to both arsenic exposure and to over-exposure to sunlight as their main occupation is farming.  As leukocytes play a major role in cure of VL, over-exposure of arsenic may restrict leishmanicidal functions of leukocytes in VL patients during treatment, leading to escape of parasites from the killing mechanism. Therefore, arsenic groundwater contamination may act as an additive risk factor for PKDL development in Bihar.  The parasites of VL generally reside in the liver and spleen tissue microenvironments and influence qualitative and quantitative aspects of the host immunity [47]. Oxidative and nitrosative stress components have serious adverse effects on the host during VL and PKDL infection. Furthermore, non-restoration ofnormal activities of peroxisomal catalase and superoxide dismutase in the host has been found responsible for unsuccessful clearance of *Leishmania* parasites from liver and spleen[48].The prolonged exposure to groundwater arsenic contamination probably adds up to the increased oxidative stress and peroxisomal dysfunction in the host. It can suggested that long time arsenic exposure may exert its influence on keratinocytes and lymphocytes, leading to modulation of cytokines that may promote development of PKDL in treated VL patients, which may also have influence on the incidence of VL in Bihar.  Our findings also suggested that there were no apparent differences between history of VL episode between the two groups of PKDL cases in the cohort (Table 1). This also indicates a probable role of environmental factors, like arsenic exposure, in PKDL development. Interestingly, the immune system has been reported to be a sensitive target for arsenic exposures that may beassociated with decreased host resistance to infectious agents [49, 50]. Arsenic causes significant changes in T-cell secreted cytokine levels with altered T-cell activation status leading to immunosuppression favoring opportunistic infections in exposed individuals. Notably, arsenitealso suppresses theactivation of Th1 (Tbet) cells, and alters the percentages of Th17 (RORγt) and Treg (FoxP3)population [51]. As T-cells are crucial deciders for the fate of VL infection, chronic arsenic exposure could also have contributed for incidence of VL. Notably, exposure to arsenic is associated with an increased prevalence of malnutrition [52], leading to susceptibility to skin lesions [53]. Malnutrition also induces immunosuppression.There is prevalence of mass malnutrition among VL patients and their family members in Bihar. Therefore, it is plausible that long term arsenic exposure could also have contributed for incidence of VL in the affected areas, finally also co-affecting PKDL incidence. However, no definite conclusions can be drawn without further studies on this aspect.  Besides possible effect of ineffective treatment of VL or other risk factors, exposure to arsenic may additionally contribute to the emergence of PKDL in Bihar, through its pro-dermatotropic effects on the parasite surface. Further work is underway to study the parasite protein expression profile during chronic arsenic exposure that would further enlighten the mechanism of parasite dermatotropism. |
| Generalisability | 21 | Discuss the generalisability (external validity) of the study results | 21 | As T-cells are crucial deciders for the fate of VL infection, chronic arsenic exposure could also have contributed for incidence of VL. Notably, exposure to arsenic is associated with an increased prevalence of malnutrition [52], leading to susceptibility to skin lesions [53]. Malnutrition also induces immunosuppression.There is prevalence of mass malnutrition among VL patients and their family members in Bihar. Therefore, it is plausible that long term arsenic exposure could also have contributed for incidence of VL in the affected areas, finally also co-affecting PKDL incidence. However, no definite conclusions can be drawn without further studies on this aspect.  Reportedly, PKDL-causing *Leishmania donovani* strains express higher levels of certain surface proteins that are associated with dermatotropism of the paraiste[54].Besides possible effect of ineffective treatment of VL or other risk factors, exposure to arsenic may additionally contribute to the emergence of PKDL in Bihar, through its pro-dermatotropic effects on the parasite surface. Further work is underway to study the parasite protein expression profile during chronic arsenic exposure that would further enlighten the mechanism of parasite dermatotropism. The current work highlights the need to consider environmental factors like arsenic exposure as an additional risk factor for PKDL development in India. However, the role of arsenic exposure on occurrence of VL cannot be ruled out. Further extensive mechanistic and epidemiological studies are required to assess the real role of arsenic exposure on PKDL development. |
| Other information | | | | |
| Funding | 22 | Give the source of funding and the role of the funders for the present study and, if applicable, for the original study on which the present article is based |  | No specific funding was involved. The study was part of the intramural research activities of the ICMR institute. |

*Give information separately for exposed and unexposed groups.

**Note:** An Explanation and Elaboration article discusses each checklist item and gives methodological background and published examples of transparent reporting. The STROBE checklist is best used in conjunction with this article (freely available on the Web sites of PLoS Medicine at http://www.plosmedicine.org/, Annals of Internal Medicine at http://www.annals.org/, and Epidemiology at http://www.epidem.com/). Information on the STROBE Initiative is available at http://www.strobe-statement.org.
